# WHAT’S IN IT? AN IN-DEPTH DESCRIPTION OF THE INDIAN COUNTRY ROAD MAP

**DOI:** 10.1093/geroni/igz038.1335

**Published:** 2019-11-08

**Authors:** Heidi Holt

**Affiliations:** Centers for Disease Control and Prevention, Atlanta, Georgia, United States

## Abstract

As tribal health and aging leaders become concerned about the growing problem of dementia, they can build on strengths in their cultures and traditions, which provide unique opportunities to improve the lives of older adults living with dementia, their families, and their communities. To offer a tool for tribal leaders, CDC, together with key partners, created The Healthy Brain Initiative: The Road Map for Indian Country. Designed to support discussion about dementia and caregiving within tribal communities, this Road Map encourages a public health approach as part of a holistic response. During this presentation, an in-depth review of the themes that shaped this Road Map will be provides, gaps in knowledge and practice will be described, and explain the 8 recommended actions in the Road Map. Discussion will wrap up with recommended actions for moving forward, as well as CDC’s plans for supporting implementation of the Road Map.

